# Gastroesophageal reflux disease: prevalence and Extraesophageal manifestations among undergraduate students in South West Nigeria

**DOI:** 10.1186/s12876-020-01292-1

**Published:** 2020-05-26

**Authors:** Moses Ayodele Akinola, Titus Ayodeji Oyedele, Kolawole Oluseyi Akande, Olukayode Yinka Oluyemi, Omotayo Felicia Salami, Alaba Moses Adesina, Adedeji David Adebajo

**Affiliations:** 1 Ben Carson School of Medicine, Babcock University, Ilishan- Remo, Ogun State Nigeria; 2 Gastroenterology Unit, Department of Medicine, College of Medicine, University of Ibadan, Ibadan, Oyo State Nigeria; 3 Olabisi Onabanjo University Teaching Hospital, Sagamu, Ogun State Nigeria

**Keywords:** Gastroesophageal reflux extraesophageal disease, Nocturnal cough, Dental erosion, Halitosis

## Abstract

**Background:**

The reflux of noxious contents of the stomach may cause oesophageal and extra-oesophageal complications either by direct contact of aspirated gastric refluxate with the upper airway or by a vago-vagal reflex. This study aimed to determine the prevalence of gastroesophageal disease (GERD) and extraesophageal manifestations among undergraduate students in a tertiary institution in Nigeria.

**Methods:**

This is a cross-sectional study involving undergraduate students in a private University in Nigeria. Study proforma had three parts. Part A consisted of self-administered questionnaire designed to obtain students biodata. Part B consisted of standard Carlsson-Dent questionnaire. A score of 4 and above on Carlsson- Dent questionnaire was considered diagnostic of gastroesophageal reflux symptoms (GERD). Thereafter those who had GERD were further questioned and examined for extra-oesophageal symptoms of GERD.

**Results:**

The total number of the study participants was 647, out of which 212 (32.8%) had GERD. One hundred and forty-four (67.9%) and 86 (32.1%) females and male had GERD respectively (*p* = 0.13).

The extraesophageal symptoms found in those with GERD were, dysphagia, coated tongue, nocturnal cough, xerostomia, lump in the throat, asthma-like symptoms, recurrent sore throat, frequent throat clearing, halithosis and dental erosion among others.

**Conclusion:**

GERD is common among this study population, with a prevalence rate of 32.8%. Only age showed significant predictor for GERD. Varying extra-oesophageal manifestations were found in those with GERD.

## Background

Gastroesophageal reflux disease (GERD) occurs due to reflux of gastric contents into the oesophagus leading to persistent symptoms and or complications [1]. Oesophageal manifestations of GERD include heartburns, regurgitation, oesophagitis, Barrett’s oesophagus and oesophageal adenocarcinoma. In addition, GERD has an array of manifestations and complications beyond the oesophagus and these are called extra-oesophageal or supra oesophageal manifestations or features of GERD [2]. The organs that are mainly involved in these extra oesophageal manifestations of GERD include lungs, Ear, Nose, Throat (ENT) and the mouth [3]. GERD manifestations in the ENT system include chronic laryngitis, chronic sinusitis, otitis media, hoarseness, cough, globus, sore throat, post nasal drip, nasal congestion, halitosis, paroxysmal laryngospasm, laryngeal and subglottic stenosis and laryngeal and pharyngeal carcinoma [4–7]. Respiratory features of GERD include chronic cough, choking episodes, aspiration pneumonitis, asthma-like disease among others. GERD is also a common cause of dental erosion which occurs as a result of long-term exposure to acid [8]. It is usually a slow process occurring over many years and its subtle appearances may be easily missed. Therefore, dental erosion is usually detected only after significant damage has occurred to the dentition and the masticatory system [9].

There is paucity of information in Nigeria regarding the GERD especially, its extraesophageal manifestations. There is therefore the need to find out the prevalence of GERD and its extraesophageal manifestations in our population.

The main objective was to determine the prevalence of gastroesophageal reflux symptoms and its extraesophageal manifestations among undergraduate students of Babcock University. Our specific objectives were to determine ear, nose, throat, respiratory and dental manifestations of GERD and the relationship between it and obesity among this study population.

## Methods

This cross-sectional study was carried out in Babcock University, a private university located in Ilishan Remo, Ogun State, South west Nigeria with a students’ population of 8000 as at the time of this study.

### Study participants

This study involved all undergraduate students at Babcock University residing in school halls of residency within the study period, who agreed to participate in the study.

### Sample size

The Sample size for this study was determined using Leslie- Kish formula for population-based study at 95% confidence level with 50.0% prevalence and degree of freedom put at 0.05%. Using the formula, the sample size was 384 including 10% attrition rate. However, for this study 800 students were targeted but 668 students participated. Others did not give consent to participate.

### Sampling technique

All the 8 students’ hostels were used for the study. Using the register of the students in each hostel, 100 students were randomly selected per hostel. The rooms of the randomly selected students were obtained from the register and they were approached in their respective rooms. Male Assistants were engaged for male hostels while female Assistants for the female hostels.

### Instrument for the study

#### The instrument for this study consisted of three parts: A, B and C

Part A was a proforma questionnaires for students’ bio data, weight, height and waist circumference while Part B consisted of Carlsson- Dent questionnaire for diagnosis of GERD^.^ Carlsson-Dent questionnaire is a 7-item questionnaire on typical symptoms of GERD and their relationship to meal, antacid use, posture, straining and effect of regurgitation. Positive or negative marks were awarded to the answers to the questions. A summation score of 4 and above was considered diagnostic of GERD [10]. Part C consisted of questionnaire on extraoesophageal symptoms developed the researchers and the Smith and Knight tooth wear index to assess dental erosion [11].

### Data collection procedure

Trained research Assistants administered part A and B of the research instrument. Each subject had weight, height, and waist circumference taken. Weight (Kg) was taken using bathroom scales while heights were taken with stadiometers. Stretch -resistant tapes were used to measure the waist circumference at a point mid- way between the last rib and the iliac crest. The Subjects who had GERD based on the Carlson-Dent questionnaire score of 4 and above were recruited for the part C of the study (extra-oesophageal symptoms and examination). They were questioned and examined by the ENT surgeon, Orthodontist and a Chest Physician.

### Data analysis

The age of the participants were grouped into 15–18 years, 19–22 years and greater or equal to 23 years, the BMI was classified as underweight (< 18 kg/m^2^), normal (18–24.9 kg/ m^2^), over-weight(25–29.9 kg/m^2^), and obese (40 > kg/m^2^). Waist circumference was also classified into normal (< 94 cm for male, < 80 cm for female), risky (94–101 cm for male, 81-88 cm for female) and highly risky (≥ 102 cm for male, ≥ 88 cm for female) waist circumference. Descriptive analysis was used to describe the demographic variables, the prevalence of GERD.

Pearson’s Chi square test was used to establish the association between GERD, age, sex, BMI and waist circumference. Inferential analysis using logistic regression was done to determine predictors of GERD. Values was be taken to be significant when *P* value was < 0.05.

### Ethical consideration

The protocol to the study was submitted to the peer review committee of Babcock University Health Research Ethics Committee-BUHREC and was subsequently approved (BUHREC 002/17). Written informed consent was taken from all the Subjects that participated in the study.

## Results

A total number of 668 students participated in the study but only 647 questionnaires were analysed. The remaining 21 questionnaires were not properly filled. Gender distribution of the study participants showed that 413 (63.8%) were females while 234 (36.2%) were males. The number of Participants with gastroesophageal reflux symptoms was 212 giving a prevalence of 32.8%.

Table 1 showed age and gender distribution of the study participants. The age group 15–18 years had the highest number of Participants with GERD (38.7%) while aged 19–22 has the least number of Participants with GERD (29.3%). The association between the age of the Participants and GERD was not statistically significant (*p* = 0.06). This study showed that there were more females with GERD (34.9%) when compared with their male counterpart (29.1%) (*p* = 0.13).

**Table 1:** Showing socio-demographic data of the study participants

	GERD				
Variables	Present	Absent	✗^**2**^	***P***-Value	Total
Age (Years)					
15–18	**84 (38.7)**	**133 (61.3)**	**5.63**	**0.06**	**217 (33.5)**
19–22	**114 (29.3)**	**275 (70.9)**			**389 (60.1)**
≥ 23	**14 (34.1)**	**27 (65.9)**			**41 (6.3)**
Gender					
Males	**68 (29.1)**	**166 (70.9)**	**2.29**	**0.13**	**234 (36.2)**
Females	**144 (34.9)**	**269 (65.1)**			**413 (63.8)**
BMI					
≤ 18.4	**22 (36.1)**	**39 (63.9)**	**2.90**	**0.41**	**61 (9.4)**
18.5–24.9	**131 (32.5)**	**272 (67.5)**			**403 (62.3)**
25–30	**38 (28.8)**	**94 (71.2)**			**132 (20.4)**
≥ 30	**21 (41.2)**	**30 (58.8)**			**51 (7.9)**
Total	**212 (32.8%**	**435 (67.2)**			**647 (100%)**
Waist circumference /gender					
Female					
Normal WC	**109 (36.2)**	**192 (63.8)**	**4.32**	**0.23**	**301 (46.5)**
Risk	**19 (27.5)**	**50 (72.5)**			**69 (10.7)**
Highly risk	**16 (37.2)**	**27 (62.8)**			**43 (6.6)**
Male					
Normal WC	**59 (29.2)**	**143 (70.8)**	**2.32**	**0.51**	**202 (31.2)**
High risk	**8 (28.6)**	**20 (71.4)**			**28 (4.3)**
Great risk	**1 (25.0)**	**3 (75.0)**			**4 (0.6)**

In addition, participants with BMI ≥30 had higher percentage of GERD (41.2%), followed by those with BMI of ≤18.4 (36.1%), then BMI of 18.5–24.9 (32.5%) while participants with BMI of 25–30 had the least GERD (28.8%). The association between BMI and GERD was also not statistically significant (*p* = 0.41). Also, there was no statistically significant association between GERD and waist circumference in both males and females (*p* = 0.51 and *p* = 0.23).

Table 2 shows the result of multivariate logistic regression to determine the predictors of GERD in the study population. Only age shows significant association with GERD, participants aged 19–22 years showed reduced odds of having GERD compared with other age groups (*p* = 0.04). Participants who were obese and underweight showed increased odds of having GERD (AOR 1.67; 95% C. I 0.84–3.33; *P* = 0.15) and (AOR 1.14; 95% C. I 0.65–2.01; *P* = 0.65) respectively. Male participants showed reduced odds of having GERD (AOR 0.77; 95% C. I 1.53–1.13; *P* = 0.19), then participants from age group greater than 23 year (AOR 0.91; 95% C. I 0.45–0.88; *P* = 0.81), overweight participants (AOR 0.90; 95% C. I 0.57–1.42; *P* = 0.66). The waist circumference was not a significant predictor of GERD in both male and female Participants.

**Table 2:** Logistic regression showing predictors of GERD in the study population

Variables	GERD		Adjusted OR	95% C.I	***P***-value
	Present	Absent			
**Female**	**144 (34.9)**	**269 (65.1)**	**1**	**–**	**–**
**Male**	**68 (29.1)**	**166 (70.9)**	**0.77**	**0.53–1.13**	**0.19**
**Age**					
**15–18**	**84 (38.7)**	**133 (61.3)**	**1**	**–**	**–**
**19–22**	**114 (29.3)**	**275 (70.9)**	**0.68**	**0.48–0.98**	**0.04**
**≥ 23**	**14 (34.1)**	**27 (65.9)**	**0.91**	**0.45–1.88**	**0.81**
**BMI**					
**Normal**	**131 (32.5)**	**272 (67.5)**	**1**	**–**	**–**
**Underweight**	**22 (36.1)**	**39 (63.9)**	**1.14**	**0.65–2.01**	**0.65**
**Overweight**	**38 (28.8)**	**94 (71.2)**	**0.90**	**0.57–1.42**	**0.66**
**Obese**	**21 (41.2)**	**30 (58.8)**	**1.67**	**0.84–3.33**	**0.15**
**Waist circumference/gender**					
**Female(≥80)**	**19 (27.5)**	**50 (72.5)**	**0.62**	**0.34–1.16**	**0.14**
**Female(≥88)**	**16 (37.2)**	**27 (62.8)**	**0.86**	**0.34–1.84**	**0.69**
**Male(≥90–101)**	**8 (28.6)**	**20 (71.4)**	**0.62**	**0.34–1.16**	**0.14**
**Male(≥102)**	**1 (25.0)**	**3 (75.0)**	**0.86**	**0.40–1.84**	**0.69**

Table 3 shows various extraesophageal symptoms seen among those with GERD. Out of 212 with GERD, 69 (32.0%) reported at the clinic for assessment on extraesophageal manifestation of GERD.

**Table 3:** Showing extra-esophageal features found among the students with GERD *N* = 69

Variables	Extra-oesophageal features		Total
	Present n (%)	Absent n (%)	N (%)
**Frequent throat clearing**	**18 (26.1)**	**51 (73.9)**	**69 (100.0%)**
**Recurrent sore throat**	**16 (23.2)**	**53 (76.8%)**	**69 (100.0%)**
**Regurgitation of recently ingested meals**	**15 (21.7)**	**54 (78.3)**	**69 (100.0%)**
**Chocking sensation on taking fluid**	**14 (20.3)**	**55 (79.7)**	**69 (100.0%)**
**Sensation of lump in the throat**	**12 (17.4)**	**57 (82.6)**	**69 (100.0%)**
**Difficulty in breathing especially in the night**	**12 (17.4)**	**57 (82.6)**	**69 (100.0%)**
**Asthma-like symptoms**	**12 (17.4)**	**57 (82.6)**	**69 (100.0%)**
**Nocturnal cough**	**11 (15.9)**	**58 (84.1)**	**69 (100.0%)**
**Dysphagia**	**7 (10.1)**	**62 (89.9)**	**69 (100.0%)**
**Chronic/recurrent cough**	**6 (8.7)**	**63 (91.3)**	**69 (100.0%)**

Out of 69 students who reported in the clinic, 8.7% had chronic or recurrent cough, 10.1% had halitosis, 10.1% had excessive phlegm, 10.1% had dysphagia, 14.5% had coated tongue, 15.9% had nocturnal cough, 17.4% had xerostomia, 17.4% had feeling of lump in the throat, 17.4% had difficulty in breathing especially at night, 17.4% had asthma-like symptoms, 20.3% had chocking sensation on taking fluid, 23.2% had recurrent sore throat, 26.1% had frequent throat clearing and 37.7% had hoarseness. Dental erosion was found 36.2% out of which 19% were mild erosion, 7.2% moderate erosion and 1.4% had severe dental erosion. See Table 4.

**Table 4:** Showing oral features found in students with GERD *N* = 69

Variables	Extra esophageal features		Total
	Present n (%)	Absent n (%)	N (%)
**Dental erosion**	**25 (36.2)**	**44 (63.8)**	**69 (100.0)**
Xerostomia	12 (17.4)	57 (82.6)	69 (100.0)
Coated tongue	10 (14.5)	59 (85.5)	69 (100.0)
Halitosis	7 (10.1)	62 (89.9)	69 (100.0)

## Discussion

In Nigeria most studies on GERD were carried out on patients referred for upper gastrointestinal endoscopy where diagnosis was limited to patients with endoscopically defined lesions [12]. However, only about 50% of patients with GERD will have endoscopically detectable lesions [13, 14]. Therefore, the use of a patient-centred, symptom driven approach to diagnosis which is independent of endoscopic findings is more appropriate. The prevalence of GERD in a similar community-based study carried out in Nigeria was 26.34% which is comparable to 32.8% reported in this study though the studies were from different geo-political zones of Nigeria [15]. Even though the use of symptom-based questionnaire is advocated in the diagnosis of GERD, there are many of such questionnaires, each with its strength and weakness [16]. We decided to use Carlsson- Dent questionnaire though not validated but has been used by two previous studies in Nigeria [15, 17]. When compared with GERD Q, another commonly used GERD questionnaire. Carlsson-Dent questionnaire was found to be easier for patients to understand and answer although it detected less GERD in patients that were overweight and obese [18]. A Thai study found Carlsson-Dent questionnaire diagnosed GERD more than PH monitoring and endoscopy although a prospective, open label multi-centre Dutch study reported a poor diagnostic performance of Carlsson-Dent [19, 20].

GERD has been associated with many factors [21]. In this study, age had a significant association with GERD with participants between the age group of 19–22 years having reduced odds of having GERD compared with age groups 15–18 years and age group greater than 23 years.

Song et al. [22] reported an increased risk for GERD in overweight and obese subjects. However, in this study we found that there are increased odds of having GERD in both underweight and obese subjects. The association between GERD and underweight is surprising and we have no explanation for it. It may however be related to spices contents of food as suggested by Song et al. [22] .

Gastroesophageal reflux disease causes extra-oesophageal symptoms by direct and indirect mechanisms. The direct is by aspiration while the indirect is vagally mediated [23, 24].

Chronic cough is a known extra-oesophageal manifestation of GERD. The most common causes of chronic cough in non-smoking patients with normal chest radiographs, who are not on angiotensin converting enzyme (ACE) inhibitors are post nasal drip syndrome (PNDS), asthma, gastroesophageal reflux and chronic bronchitis [25]. In our study, 8.7% of the study population had chronic or recurrent cough while 15.9% had nocturnal cough. Poe et- al found that GERD alone accounted for cough in 13% of their study population, while in 56% of patients, it was a contributing factor to persistence of cough [26].

Gastroesophageal reflux disease is a known aetiology of laryngeal inflammation otherwise called laryngopharyngeal reflux (LPR) [27]. Symptoms of LPR include hoarseness, throat pain, cough, hawking, dysphagia, odynophagia and voice fatigue. However, these symptoms are nonspecific and can also be seen in other patients with postnasal drip and those exposed to allergens and smoke. In our study population, 37.7% of students with GERD had hoarseness and 10.1% of them had dysphagia. Dysphagia in these group of patients is likely due to GERD as there are no demographic or clinical features suggestive of other oesophageal diseases like carcinoma.

Asthma has a strong correlation with GERD and the conditions seem to induce each other. Both epidemiologic studies and physiologic testing with ambulatory 24-h pH monitoring have established association between GERD and asthma [27, 28]. In our study we found asthma-like symptoms in 17.4% of students with GERD. In studying the prevalence of GERD in asthma patients, Kijander et al. found that though 35% of GERD related patients did not express the typical reflux symptoms, they had abnormal oesophageal acid exposure by pH monitoring [29] Similarly, Legget et al. conducted a study assessing GERD in patients whose asthma was difficult to control using 24-h ambulatory pH probes [30] .They reported that 55% had reflux at the distal probe while 35% had proximal probe reflux [31]. They therefore submitted that reflux occurs commonly in asthma patients.

This study also showed that dental erosion is a significant finding in subjects with GERD. A larger percentage of study participants in this study had dental erosion which is due to the gastric acidity [32]. ^.^ A study by Oginni et al. [33] reported that tooth wear index (TWI) scores were higher in patients with GERD than in control subjects. The frequency of regurgitation and duration of gastroesophageal reflux directly influence the severity of dental erosion.

In addition, other oral features such coated tongue, Xerostomia and halitosis were reported in some of the study participants with GERD. Impaired lower esophageal sphincter function also results in gas and stomach contents entering the esophagus resulting in halitosis.

Our study has some limitations. Information on the lifestyles of the Participants were not collected in this study because two other studies had focused on GERD, diet and lifestyles in our population [15, 17] and we felt there was no need for repetition. Secondly, Participants in this study, being University students, cannot be said to be representative of the Nigerian population. Lastly, we studied extra-oesophageal manifestations in those diagnosed with GERD only. Extra oesophageal symptoms of GERD have been reported without the typical symptoms of heartburns and or GERD [34]. Though there is no gold standard test for the association between extraoesophageal symptom and GERD, it is necessary, in our opinion, to do oesophageal PH monitoring and or GI endoscopy with oesophageal biopsy to be able to attribute those features to GERD since these extra oesophageal symptoms are not pathognomonic of GERD [28]. In this study, we did not do either of oesophageal PH monitoring or upper GI endoscopy for the Participants.

## Conclusion

This study reported GERD prevalence of 32.8% and it identified age as the only predictor of GERD among the participants. Extra oesophageal features are quite common among the participants with GERD.

## Data Availability

The data sets used and /or analysis during the current study are available from the corresponding author on request.

## Abbreviations

GERD: Gastro Esophageal Reflux Disease
BMI: Body Mass Index
LPR: Laryngopharyngeal Reflux
ACE: Angoitensin Converting Enzyme
PNDS: Post Nasal Drip Syndrome
BUHREC: Babcock University Health Research Ethics Committee
